# Diagnosis and Subclassification of Acute Lymphoblastic Leukemia

**DOI:** 10.4084/MJHID.2014.073

**Published:** 2014-11-01

**Authors:** Sabina Chiaretti, Gina Zini, Renato Bassan

**Affiliations:** 1 Division of Hematology, Department of Cellular Biotechnologies and Hematology, “Sapienza” University of Rome, Rome, Italy; 2 Hematology, Catholic University Sacred Heart Policlinico Gemelli, Rome, Italy; 3 Hematology and Bone Marrow Transplant Unit, Ospedale dell’Angelo e SS. Giovanni e Paolo, Mestre-Venezia, Italy

## Abstract

Acute lymphoblastic leukemia (ALL) is a disseminated malignancy of B- or T-lymphoblasts which imposes a rapid and accurate diagnostic process to support an optimal risk-oriented therapy and thus increase the curability rate. The need for a precise diagnostic algorithm is underlined by the awareness that both ALL therapy and related success rates may vary greatly between ALL subsets, from standard chemotherapy in patients with standard-risk ALL, to allotransplantation (SCT) and targeted therapy in high-risk patients and cases expressing suitable biological targets, respectively. This review summarizes how best to identify ALL and the most relevant ALL subsets.

## Introduction

Current standards for acute lymphoblastic leukemia (ALL) diagnosis integrate the study of cell morphology, immunophenotype and genetics/cytogenetics as detailed in the 2008 WHO classification of lymphoid neoplasms.^1^ The classification originally suggested by the FAB group is no longer followed.^2^,^3^ The FAB classification was clinically useful since it permitted recognition of probable Burkitt lymphoma in leukemic phase, but it has now been replaced by the WHO classification. Lymphoid neoplasms are assigned, in the most recent WHO classification, to two principal categories: neoplasms derived from B- and T-lineage lymphoid precursors and those derived from mature B, T or NK cells. ALL belongs to the first of these major groups, designated B- or T-lymphoblastic leukemia/lymphoma^4^ and including three principal categories: B-lymphoblastic leukemia/lymphoma not otherwise specified, B-lymphoblastic leukemia/lymphoma with recurrent cytogenetic alterations and T-lymphoblastic leukemia/lymphoma. The designation of leukemia/lymphoma reflects the principle that these neoplasms should be classified on the basis of their biological and molecular characteristics, regardless of the sites of involvement. The leukemic variant shows diffuse involvement of the peripheral blood and the bone marrow, while lymphoma is confined to nodal or extranodal sites, with no or minimal involvement of the bone marrow. In the leukemic form, by definition, the bone marrow must contain at least 20% blast cells. A purely leukemic presentation is most typical of B-lineage ALL (85%), while cases of T-lineage disease often present with an associated lymphomatous mass in the mediastinum or other sites.

## Diagnostic Morphology and Cytochemistry

A morphological bone marrow assessment represents the first step in the diagnostic pathway, for the primary diagnosis of ALL and for the differentiation from acute myeloid leukemia (AML),^5^ since ALL, by definition, always presents with bone marrow involvement. Table 1^6^ shows the morphological criteria that are useful for distinguishing between myeloblasts and lymphoblasts, however remembering the limits of morphology in ALL, for which flow cytometry analysis represents the diagnostic gold standard for both the identification of cell lineage and the definition of subset. The morphology of leukemic cells in the peripheral blood can be significantly different from that of the bone marrow, which is always indispensable.

From the morphological point of view, there are no reproducible criteria to distinguish between B- and T-lineage ALL. It can also be difficult to distinguish B-lineage lymphoblasts from normal B-lineage lymphoid precursors, known as hematogones, which are observed in the peripheral blood in various conditions, including primary myelofibrosis and in children in the phase of recovery following chemotherapy*.*^7^ Hematogones typically have an even higher nucleocytoplasmic ratio than lymphoblasts, with more homogeneous chromatin and a complete absence of visible nucleoli. Hematogones can also express the CD10 antigen, but can be distinguished from blast cells of B ALL by other immunophenotypic features, being characterised by regular, orderly acquisition and loss of B-lineage antigens; they can also be distinguished from mature lymphocytes by their weak expression of CD45 and, sometimes, by the expression of CD34.^7^

**Figure 1:**
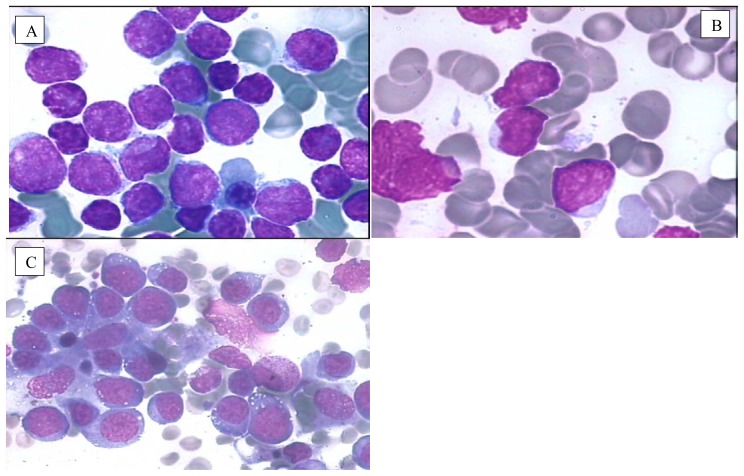
Common morphological variants of ALL. **A**) FAB L1 subtype: the lymphoblasts are small and the nuclear and cytoplasmic characteristics appear uniform with scant blue cytoplasm, regular nuclear shape, partially condensed chromatin with barely visible nucleoli and high nucleocytoplasmic ratio; **B**) FAB L2 subtype: the lymphoblasts are variable in size with irregular nuclear outlines, heterogeneous lacy chromatin, moderately plentiful weakly basophilic cytoplasm and variable nucleocytoplasmic ratio; **C**) FAB L3 subtype (Burkitt): the lymphoblasts are very large and quite homogeneous with finely granular stippled nuclear chromatin with prominent nucleoli. The cytoplasm is midnight blue and is vacuolated; the majority of such cases are now recognised as representing non-Hodgkin lymphoma rather than ALL. **B**) in this picture are displayed many lymphoblasts with ALL-L2 morphology and one lymphoblast (right side) with coarse azurophilic cytoplasmic granules.

**Figure 2:**
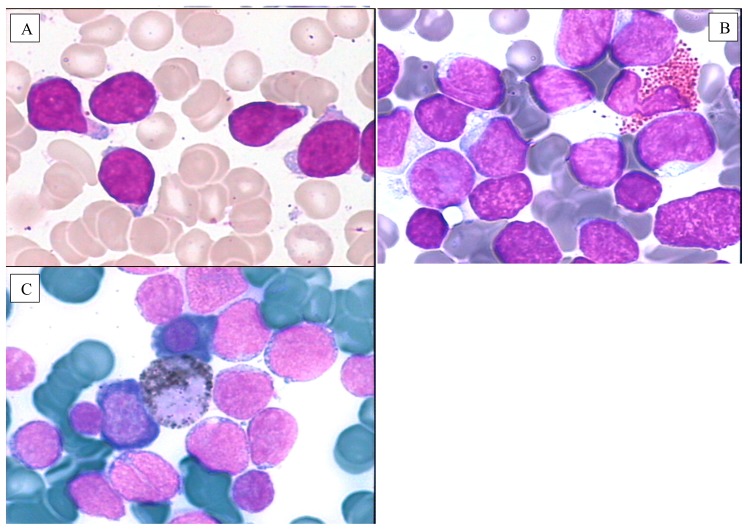
Rare morphological variants of ALL and MPO negativity. **A**) ALL with hand-mirror cells: the shape of the elongated lymphoblasts resembles a hand mirror or a tennis racquet with a very high nucleocytoplasmic ratio. Almost all the blasts have a small polar cytoplasmic projection corresponding to a uropod; **B**) Eosinophil-associated ALL; **C**) Negative MPO reaction in ALL: a yellow-brown precipitate is visible only in a neutrophil metamyelocyte just to the center of the picture. All the other blast cells are completely peroxidase-negative.

The bone marrow morphology of ALL is however quite variable as previously indicated in the FAB classification (Figures 1–2). Rare morphological variants are: ALL with “hand-mirror cells”, i.e. the shape of the cells resembles a hand mirror or a tennis racquet (Figure 2A); granular ALL, with presence of azurophilic cytoplasmic granules which vary in number, size and shape. Cytochemically, these blasts have negative peroxidase reactions and variable periodic acid-Schiff (PAS) positivity; Sudan black B is sometimes weakly positive;^8^ ALL with mature cells that are nearly indistinguishable from mature lymphoid neoplasms and require expert observers for accurate morphological identification;^9^ ALL associated with hypereosinophilia (Figure 2B). By definition, ALL blasts are negative for myeloperoxidase (MPO) **(**Figure 2C) and other myeloid cytochemical reactions. According to the FAB criteria, acute (leukemias with at least 3% MPO-positive blasts in BM should be classified as myeloid. However, low level MPO positivity without expression of other myeloid markers is detectable by means of electron microscopy in rare ALL cases. True MPO+ ALL is discussed below in the mixed lineage acute leukemias section. The acid phosphatase reaction correlates with the lysosome content; it is useful for identifying TALL blasts which show focal paranuclear positivity in more than 80% of cases. Lymphoblasts may react with non-specific esterases with a strong positivity in the Golgi zone with variable inhibition with sodium fluoride. The B lymphoblasts in FAB L3/Burkitt ALL show an intense cytoplasmic positivity to methyl green pyronine, while the vacuoles stain strongly with Oil red O, thus demonstrating their lipid content. The role of cytochemistry in differentiating ALL from AML is limited and is mainly of historical interest, since these tests have now been superseded by the far more objective results provided by the immunophenotyping.

## Diagnostic Immunophenotype

Immunophenotyping by means of multi-channel flow cytometry (MFC) has become the standard procedure for ALL diagnosis and subclassification, and was also developed as useful tool for the detection and monitoring of minimal residual disease (MRD, reviewed elsewhere in this issue). The consensus by European Group for the Immunological characterization of leukaemias (EGIL) is that a threshold of 20% should be used to define a positive reaction of blast cells to a given monoclonal antibody, except for MPO, CD3, CD79a and TdT, which are considered positive at the 10% level of expression.^10^,^11^ More recently, novel MFC strategies were developed by the EuroFlow consortium to ensure accurate methodologies through all MFC steps, in order to guarantee the reproducibility of diagnostic tests.^12^,^13^ To summarize the diagnostic issue, roughly 75–80% of cases of adult ALL are of B-cell lineage and 20–25% belong to the T-cell lineage.

## Immunophenotype of B-lineage ALL

**Figure 3:**
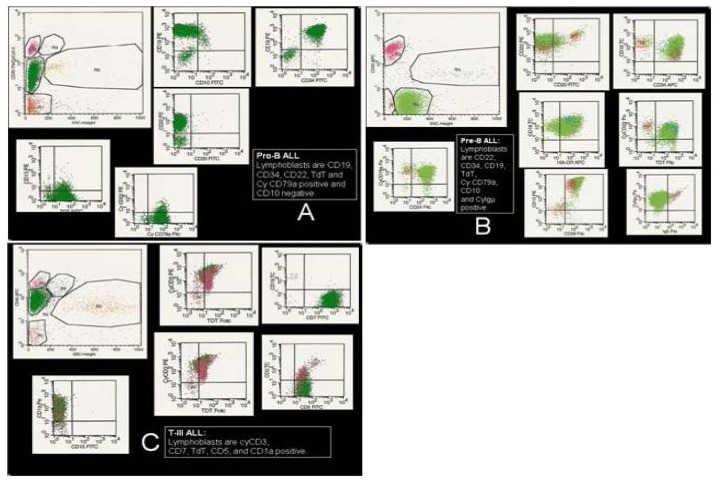
Examples of ALL immunophenotype. **A)** Pro-B ALL: lymphoblasts are CD19, CD34, CD22, TdT and Cy CD79a positive and CD10 negative; **B)** Pre-B ALL: lymphoblasts are CD22, CD34, CD19, TdT, cytoplasmic (Cy)CD79a, CD10 and Cy mμ positive; **C)** Cortical/thymic TALL: Lymphoblasts are cyCD3, CD7, TdT, CD5, and CD1a positive.

In B-lineage ALL the most important markers for diagnosis, differential diagnosis and subclassification are CD19, CD20, CD22, CD24, and CD79a. The earliest B-lineage markers are CD19, CD22 (membrane and cytoplasm) and CD79a.^14^,^15^ A positive reaction for any two of these three markers, without further differentiation markers, identifies pro-B ALL (EGIL B-I subtype) (Figure 3A). The presence of CD10 antigen (CALLA) defines the “common” ALL subgroup (EGIL B-II subtype). Cases with additional identification of cytoplasmic heavy mu chain constitute the pre-B group (EGIL B-III subtype) (Figure 3B), whereas the presence of surface immunoglobulin light chains defines mature B-ALL (EGIL B-IV subtype).

Among other B-cell markers, B-I and B-II ALL are often CD24 positive and 4G7 (pro- and pre-B surrogate light chain specific MoAb) positive;^16^ surface CD20 and CD22 are variably positive beyond stage B-I; CD13 and CD33 myeloid/cross lineage antigen can be expressed, as well as the CD34 stem cell antigen, particularly in Ph+ (Philadelphia chromosome-positive) ALL (often B-II with CD34, CD38, CD25 and CD13/33), but myeloid-specific CD117 should not be present and can be used to differentiate further between ALL and rare myeloid leukaemias with negative MPO expression. Pro-B ALL with t(4;11)/MLL rearrangements is most often myeloid antigen-positive disease (including expression of CD15). TdT expression is usually lost in B-IV subgroup. T-cell markers are usually not expressed in B-lineage ALL but a CD19+ subset is concurrently CD2+. Loss of surface adhesion molecules has been described.^17^

## Immunophenotype of T-lineage ALL

T-cell ALL constitutes approximately 25% of all adult cases of ALL. T-cell markers are CD1a, CD2, CD3 (membrane and cytoplasm), CD4, CD5, CD7 and CD8. CD2, CD5 and CD7 antigens are markers of the most immature T-cell cells, but none of them is absolutely lineage-specific, so that the unequivocal diagnosis of T-ALL rests on the demonstration of surface/cytoplasmic CD3. In T-ALL the expression of CD10 is quite common (25%) and not specific; CD34 and myeloid antigens CD13 and/or CD33 can be expressed too. Recognized T-ALL subsets are the following: pro-T EGIL T-I (cCD3+, CD7+), pre-T EGIL T-II (cCD3+, CD7+ and CD5/CD2+), cortical T EGIL T-III (cCD3+, Cd1a+, sCD3+/−) and mature-T EGIL T-IV (cCD3+, sCD3+, CD1a−). Finally, a novel subgroup that was recently characterized is represented by the so called ETP-ALL (Early-T Precursor), which shows characteristic immunophenotypic features, namely lack of CD1a and CD8 expression, weak CD5 expression, and expression of at least one myeloid and/or stem cell marker.^18^

## Mixed Phenotype Acute Leukemia

With currently refined diagnostic techniques the occurrence of acute leukemia of ambiguous cell lineage, i.e. mixed phenotype acute leukemia (MPAL) is relatively rare (<4%).^19^ These cases express one of the following feature: 1) coexistence of two separate blast cell populations (i.e. T- or B-cell ALL plus either myeloid or monocytic blast cells, 2) single leukemic population of blast cells co-expressing B- or T-cell antigens and myeloid antigens, 3) same plus expression of monocytic antigens. For myelo-monocytic lineage useful diagnostic antigens are MPO or nonspecific esterase, CD11c, CD14, CD64 and lysozyme; for B-lineage CD19 plus CD79a, cytoplasmic CD22 and CD10 (one or two of the latter according to staining intensity of CD19) and for T-lineage cytoplasmic or surface CD3. Recognized entities include Ph+ MPAL (B/myeloid or rarely T/myeloid), t(v;11q23;MLL rearranged MPAL, and genetically uncharacterized B or T/myeloid MPAL. Very rare cases express trilineage involvement (B/T/myeloid). Lack of lineage-specific antigens (MPO, cCD3, cCD22) is observed in the ultra-rare acute undifferentiated leukemia. In a recent review of 100 such cases,^20^ 59% were B/myeloid, 35% T/myeloid, 4% B/T lymphoid and 2% B/T/myeloid. Outcome was overall better following ALL rather than AML therapy.

## NK Cell ALL

CD56, a marker of natural killer (NK) cell differentiation, defines a rare subgroup of about 3% of adult ALL cases which often display other early T-cell antigens, CD7 CD2 CD5, and sometimes cCD3.^19^ True NK ALL is very rare (TdT+, CD56+, other T markers negative, unrearranged TCR genes).^21^ This diagnosis rely on the demonstration of early NK-specific CD94 or CD161 antigens.

## Differential Diagnosis

With few exceptions, ALL is readily identified by morphological marrow assessment and MFC evaluation, with no need for additional tests, since genetics/cytogenetics and genomics are available at a later stage and cannot be employed for purely diagnostic purposes, even if they add very useful clinical-prognostic information. Differentiation between ALL and AML is initially obtained by excluding reactivity to SBB or MPO stains in ALL cells (<3% positive). On cytochemical evaluation, some rare ALL cases are SBB positive but MPO and chloroacetate esterase are negative. True ALL cases that are immunoreactive to MPO or express detectable levels of MPO mRNA have been described. This can occur in Ph+ ALL and occasionally in T-lineage ALL.^22^ Evaluation of CD117 antigen expression should also be carried out.^23^ Most ALL cases express the nuclear enzyme Terminal deoxynucleotidyl Transferase (TdT). TdT-negative ALL is uncommonly reported, more in T-ALL, while it is a rule in L3/Burkitt leukemia. Therefore all TdT-negative B-precursor ALL cases must be thoroughly investigated to exclude other aggressive lymphoid neoplasms with leukemic presentation (blastic mantle cell lymphoma, atypical plasmablastic myeloma, other high-grade lymphomas).^24^,^25^

## Diagnostic Cytogenetics

Cytogenetics represents an important step in ALL classification. Conventional karyotyping can be helpful in the identification of recurrent translocations, as well as gain and loss of gross chromosomal material; however, the major limitation of this technique is that in some cases leukemic cells fail to enter metaphase. However, fluorescence in situ hybridization (FISH) can enable the detection and direct visualization of virtually all investigated chromosomal abnormalities in ALL, with a sensitivity of around 99%. Finally, array-comparative genomic hybridization (array-CGH, a-CGH) and single nucleotide polymorphisms (SNP) arrays can permit the identification of cryptic and/or submicroscopic changes in the genome. Karyotypic changes found in ALL include both numerical and structural alterations which have profound prognostic significance.^26^–^30^ With these premises in mind, the karyotypic changes that occur in ALL can be roughly subdivided in those associated respectively with a relatively good, intermediate and poor prognosis (Table 2).^31^–^34^ However, it must be kept in mind that the incidence of certain aberrations is very low, and that for some of them, the prognostic impact can be strongly affected by the type and intensiveness of therapy administered.

## Cytogenetic/Genetic Risk Groups

Among the good prognosis aberrations, it is worth mentioning del(12p) or t(12p)/t(12;21)(p13;q22) in B-lineage ALL, and t(10;14)(q24;q11) in T-ALL. These abnormalities are relatively rare in adults compared with childhood ALL.

Aberrations associated with an intermediate-risk comprise the normal diploid subset plus cases with hyperdiploidy and several other recurrent or random chromosomal abnormalities.

Other aberrations, i.e. those with isolated trisomy 21, trisomy 8, and perhaps del(6q) and t(1;19)(q23;p13)/E2A-PBX1 may constitute an intermediate-high risk group; recent evidence suggests that the dismal outcome previously reported for the t(1;19)(q23;p13)/E2A-PBX1 is overcome by current therapeutic approaches.^35^,^36^ Other recently identified aberrations in the intermediate high-risk group are represented by iAMP21^37^ and IGH rearrangements, including *CRLF2*.^38^

Finally, patients with t(9;22)(q34;q11)or BCR-ABL1 rearrangements or a positive FISH test (Ph+ ALL), t(4;11)(q21;q23) or MLL rearrangements at 11q23, monosomy 7, hypodiploidy/low hypodiploidy (and the strictly related near triploid group) fall into the poor-risk cytogenetic category, with an overall disease-free survival (DFS) rate of about 25%, or 10% in the case of Ph+ ALL prior to the introduction of tyrosine kinase inhibitors (TKI).^39^–^42^ Ph+ ALL may constitute 25–50% of CD10+ common or pre-B ALL cases and represent the most frequent abnormality in the adult/elderly, being detected in more than 50% of cases in 6^th^ decade of life.^43^ Secondary chromosome abnormalities in addition to t(9;22)(q34;q11) may worsen the prognosis;^44^ however, this is as yet unproven in TKI era.^45^ Currently, the most unfavorable group within cases with known genetic/molecular aberration is represented by t(4;11)(q21;q23) + *MLL1*-rearranged ALL, for which outcome is very poor unless allogeneic transplantation is adopted.^46^

Some other karyotypes are unique to specific ALL syndromes. Translocations involving chromosome 8 *(MYC* gene), such as t(8;14)(q24;q32) (90% of cases), t(8;22)(q24;q11)(10% of cases), and t(2;8) (rarely observed), are virtually present in 100% of cases of mature B-ALL with L3/Burkitt morphology and clonal surface immunoglobulins. Typical cytogenetic aberrations are also found in T-lineage ALL.^47^ The most frequent involve 14q11 breakpoints e.g. t(10;14)(q24;q11), t(11;14)(p13;q11), or other. The presence of t(8;14) with breakpoints at q24;q11 (q24;q32 in B-ALL) in T-ALL is associated with a lymphomatous, aggressive presentation.^48^,^49^

## New Genetics and Genomics in ALL

The integration of results of several techniques, i.e. gene expression profiling (GEP), SNP array analysis, and currently next-generation sequencing (NGS), have permitted a better definition of the molecular scenario of ALL and the identification of a constellation of novel mutations; as for the latter, however, caution must be shown, since while the biological role has been elucidated for some, while further investigation is required for others. These findings are detailed below (Tables 3, 4).

### B-lineage ALL

*IKZF1*, encoding for the transcription factor Ikaros, is frequently disrupted in BCR/ABL+ ALL (80% of cases). *IKZF1* deletions, that can be different in size, are predictors of poor outcome in Ph+ ALL,^50^–^52^ as well as in non -Ph+ ALL.^53^–^55^

Deregulated overexpression of *CRLF2* (Δ–CRLF2), found exclusively in 5–10% B-ALL cases without known molecular rearrangements^56^,^57^ is usually sustained by two types of aberrations: a rearrangement that involves *CRLF2* and the Ig heavy chain locus (IGH@-CRLF2) or an interstitial PAR1 deletion that juxtaposes intron 1 of *P2RY8* to the coding region of *CRLF2* itself. More rarely, *CRLF2* mutations can be detected. Δ-CRLF2 can be detected together with *IKZF1* deletion in Ph-negative ALL patients and with *JAK* mutations (*JAK1* or *JAK2*) or *IL7R* mutations; furthermore, they are identified in roughly 50% of children with Down syndrome;^55^,^58^ although some contrasting results have been reported, its presence correlates with an overall poor outcome.^54^,^55^

By the integration of genome -wide technologies, the “BCR/ABL-like” subgroup has been suggested/identified in both the adult^59^,^60^ and pediatric populations^61^,^62^ and it accounts for about 15% of BALL cases. This subgroup is characterized by a gene expression signature that is similar to that of BCR/ABL+ patients, frequent detection of *IKZF1* deletions and *CRLF2* rearrangements and adismal outcome. NGS has revealed the presence of mutations and/or rearrangements activating tyrosine kinases, i.e *IGH-CRLF2*, *NUP214-ABL1* rearrangements, in-frame fusions of *EBF1-PDGFRB*, *BCR-JAK2* or *STRN3-JAK2* and cryptic *IGH-EPOR* rearrangements.^63^ The recognition of this subgroup is of relevance, because of the poor prognosis observed. Open issues are represented by difficulty in detecting them with techniques other than gene expression profiling, which is not routinely performed in all centers, and by the fact that there is not a recurrent common lesion underlying the signature identified. With this in mind, it is plausible that the use of TKIs and/or mTOR inhibitors might be of benefit in these patients, as suggested by xenograft models.^64^,^65^

Hypodiploid ALL, regarded as a poor prognosis group, has been extensively evaluated in pediatric ALL:^66^ NGS proved that lesions involving receptor tyrosine kinases and RAS signaling (i.e. *NRAS*, *KRAS*, *FLT3* and *NF1*) can be detected in up to 70% of near haploid cases, whereas low hypodiploid cases are characterized by lesions involving members of the Ikaros family, particularly *IKZF2*, and by *TP53* disruptions, that can be identified in 91.2% of these cases. In adult ALL, these cases are characterized by nonrandom chromosomal losses and the *CDKN2A/B* locus deletion as sole recurrent abnormality; as already reported in children, these cases frequently harbor *TP53* mutations.^67^

*TP53* disruption has been also recently evaluated in childhood and adult ALL. In children^68^–^71^ this is detected in 6.4% and 11.1% of relapsed B-ALL and TALL cases, and, in a smaller minority of cases, also at diagnosis. A correlation with poorer outcome has been shown. In adults, *TP53* mutations are identified at diagnosis in 8.2% of cases (11.1% T-ALL and 6.4% BALL), and are preferentially identified in cases without molecular aberrations, where they are detected in 14% of cases, and are associated with refractoriness to chemotherapy.

Other lesions identified by NGS in B-lineage ALL, are represented by mutations in *CREBBP* and its paralogue, *EP300* (p300),^72^ which were identified in the relapse samples and appear to be more frequent in hyperdiploid relapsed cases.^73^ Similarly, *NT5C2* mutations, which confer increased enzymatic activity on the NT5C2 protein, which normally dephosphorylates nucleoside analogs, such as mercaptopurine, used in consolidation and maintenance therapy, have been described. 74 Results are summarized in Table 3.

### T-lineage ALL

In T-ALL, well-recognized aberrations include the T-cell receptor (TCR) gene rearrangements, chromosomal deletions, and focal gene deletions (Table 4).^75^–^83^ Moreover, chromosomal rearrangements can also lead to in-frame fusion genes encoding chimeric proteins with oncogenic properties such as *PICALM-MLLT10*, *NUP214-ABL1* fusion formed on episomes, *EML-ABL1*, *SET-NUP214* fusion and MLL gene rearrangements with numerous different partners. The prognostic significance of these lesions is uncertain.

Furthermore, the ETP subgroup and/or myeloid-like subgroup emerged as a grey zone between AML and TALL by applying genome-wide technologies.^18^,^84^,^85^ Initially, the reported incidence of this subgroup was established at around 10% of T-ALL cases; however, with the better recognition of these cases, its frequency is likely to be higher. Immunophenotype is characterized by an early T-cell phenotype and co-expression of at least one myeloid marker, while at the transcriptional level they have a stem-cell like profile with overexpression of myeloid transcription factors (including *CEBPA*, *CEBPB*, *CEBPD*), and a set of micro-RNAs (miR-221, miR-222 and miR-223). NGS has highlighted the presence of mutations usually found in acute myeloid leukemia (*IDH1*, *IDH2*, *DNMT3A*, *FLT3* and *NRAS*),^86^ as well mutations in the *ETV6* gene. Finally, these cases rarely harbor *NOTCH1* mutations.^87^ Overall, prognosis is poor in these cases.

A large set of mutations (Table 4) has been identified in T-ALL by re-sequencing and NGS: they include *NOTCH1*, *FBW7*, *BCL11B*, *JAK1*, *PTPN2*, *IL7R* and *PHF6*, beyond those identified in ETPs; some of them have recognized prognostic significance, whereas for others further studies are required. In fact, *NOTCH1* and/or *FBW7* mutations, which occur in more than 60% and about 20% of cases, respectively, are usually associated with a favorable outcome. In the light of this, a prognostic model has been recently proposed, defining as low-risk patients those who harbor *NOTCH1* and FBW7 mutations, and as high risk those without these mutations or with lesions involving *RAS/PTEN*.^83^,^88^–^91^ At variance, *JAK1* mutations, which increase JAK activity and alter proliferation and survival have been associated with chemotherapy refractoriness and should be considered as poor prognostic markers.^92^–^94^

Finally, another group of mutations/lesions is possibly involved in leukemogenesis, but their prognostic impact is either unknown or absent. They include: 1) *BCL11B* lesions, which can induce a developmental arrest and aberrant self-renewal activity;^95^,^96^ 2) *PTPN2*- a negative regulator of tyrosine kinases-, mutations, often detected in TLX1 overexpressing cases, T-ALL, NUP214-ABL+ patients and *JAK1* mutated cases;^97^,^98^ 3) mutations in *IL7Ralpha*, that lead to constitutive JAK1 and JAK3 activation and enhancement of cell cycle progression;^99^,^100^ 4) *PHF6* mutations; 101,1025) mutations in *PTPRC*, encoding the protein tyrosine phosphatase CD45, usually detected in combination with activating mutations of *IL7R*, *JAK1* or *LCK*, and associated with downregulation of CD45 expression;^103^ 6) mutations in *CNOT3*, presumed to be a tumor suppressor; 7) mutations of *RPL5* and *RPL10*, which impair ribosomal activity.^104^ Lastly, similarly to what is observed in relapsed B-ALL, *NT5C2* mutations. 105

## Concluding Remarks

**Figure 4:**
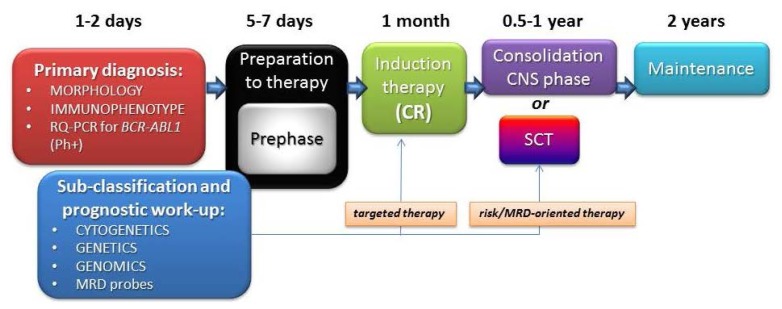
Diagnosis and subclassification of adult ALL. To confirm diagnosis and obtain clinically useful information, it is necessary to 1) differentiate rapidly Ph-positive ALL from Ph-negative ALL in order to allow an early introduction of tyrosine kinase inhibitors in the former subset, 2) distinguish between different clinico-prognostic Ph- ALL subsets, and 3) clarify diagnostic issues related to the application of targeted therapy and risk-/minimal residual disease (MRD)-oriented therapy. The early diagnostic phase must be completed within 24–48 hours. Additional test for cytogenetics/genetics, genomics and MRD rely on collection, storage and analysis of large amounts of diagnostic material, and are usually available at later time-points during therapy, however before taking a decision for allogeneic stem cell transplantation (SCT). All this requires a dedicated laboratory, and is best performed within a prospective, well coordinated clinical trial.

Due to the reviewed evidence and the complexity of all the issues at play, it is recommended that adult patients with ALL should be treated within prospective clinical trials, which is the best way to ensure both diagnostic accuracy and therapeutic efficacy. In the context of a modern risk- and subset-oriented therapy, the early diagnostic work-up is of the utmost importance and therefore needs to be carried out by well trained and highly experienced personnel (Figure 4). As a first step, it is mandatory to differentiate rapidly Ph+ from Ph-ALL and to distinguish between major immunophenotypic subsets in the latter group. The remaining diagnostic elements are available at a later stage and permit a proper identification and treatment of the several disease and risk entities. Ongoing research will permit the further definition of novel subgroups with prognostic significance.

## Figures and Tables

**Table 1:** Morphological characteristics of blasts cells in acute lymphoblastic leukemia versus acute myeloid leukemia (adapted from Morphology of Blood Disorders, 2nd Edition. d’Onofrio G, Zini G, Bain B.J. 2014.)

	Lymphoblasts	Myeloblasts
General characteristics	Blast population tends to be homogeneous	Blast population tends to be heterogeneous, with the exception of the undifferentiated form
Size	Variable, mainly small	Variable, mainly large
Nucleus	Central, mainly round; sometimes indented, particularly in the form in adults Nucleocytoplasmic ratio very high in the form that occurs in children Nucleocytoplasmic ratio lower in the form that occurs in adults	Tending to be eccentric, round, oval or angulated; sometimes convoluted, particularly in the form with a monocytic component Nucleocytoplasmic ratio high in undifferentiated blast cells and in some megakaryoblasts Nucleocytoplasmic ratio mainly low in the form with differentiation
Chromatin	Fine, with dispersed condensation Very condensed in small lymphoblasts	Fine, granular, delicately dispersed
Nucleoli	Absent in small lymphoblasts Sometimes indistinct	Almost always present, often large and prominent, double or triple
Cytoplasm	Scanty, basophilic Sometimes with a single long projection (‘hand-mirror cell’)	Variable Abundant in monoblasts With protrusions in erythroblasts and megakaryoblasts
Granules	Rarely present, azurophilic and always negative for peroxidase, esterases and toluidine blue	Present in forms with differentiation and positive with cytochemical stains – peroxidase in the neutrophil and esoinophil lineages –nonspecific esterase in the monocyte lineage –toluidine blue in the basophil lineage
Auer rods	Always absent	Can be present Typically present in the hypergranular promyelocytic form
Vacuolation	Can be present	Can be present Almost always present in forms with a monocytic component

**Table 2:** Cytogenetics and prognosis in Ph-negative ALL. Two karyotype-related prognostic classifications of Ph-negative ALL, as derived from two recent clinical series (**31,32**). Definition of risk groups is according to the SWOG study, ranging from <30% for the very high risk group to 50% and greater for the favorable subtypes. Some differences are observed in the normal and “other” karyotypic subgroups, which are assigned to the next better category in the SWOG study compared to MRC-ECOG. It is necessary to note that 9p deletions are not always associated with a favorable prognosis. In a study identifying 18 such cases, survival was short and comparable to Ph+ ALL (**33**).

	MRC-ECOG (N = 1366)*		SWOG (N = 200)**	

Cytogenetic risk group	No. (%)	5-year OS probability	No. (%)	5-year OS probability

**Favorable** *(OS >50%):*				0.52^1^
del(9p)	71 (9)	0.58	3 (2)	
high hyperdiploid	77 (10)	0.53	1 (<1)^2^	
low hyperdiploid	-	-	6 (4)	
tetraploid	15 (2)	0.65	-	
	
**Intermediate** *(OS 40–50%):*				
t(10;14)	16 (2)	0.41	-	
abn 11q	29 (4)	0.48	-	
del(12p)	29 (4)	0.41	-	
del(13q)/−13	40 (5)	0.41	-	
normal	195 (25)	0.48	31 (22)	
other	-	-	32 (23)	

**High** *(OS 30–40%):*				0.47^3^
del(6q)	55 (7)	0.36	-	
−7	19 (2)	0.36	1 (<1)	
del(7p)	-	-	2 (1)	
del(17p)	40 (5)	0.36	-	
other 11q23	15 (2)	0.33	2 (1)	
t(1,19)	24 (3)	0.32	7 (5)	
other TCR	18 (2)	0.33	-	
14q32	45 (6)	0.35	-	
Other	102 (13)	0.39	-	

**Very high** *(OS <30 %):*				0.22
t(4;11)	54 (7)	0.24	6 (4)	
t(8;14)	16 (2)	0.13	-	
del(7p)	23 (3)	0.26	-	
+8	23 (3)	0.22	-	
+X	34 (4)	0.27	-	
complex	41 (5)	0.28	12 (9)	
low hypodiploid/near triploid	31 (4)	0.22	1 (<1)	

OS, overall survival; low hyperdiploid: 47–50 chromosomes; high hyperdiploid: 51–65 chromosomes; tetraploid: >80 chromosomes; low hypodiploid: 30–39 chromosomes; near triploid: 60–78 chromosomes; complex: >5 unrelated clonal abnormalities

* evaluable N = 1003; 267/1373 (19%) evaluable by cytogenetics/FISH/RT-PCR had Ph+ ALL and were excluded from analysis (5-year OS probability 0.22)

** evaluable N = 140; 36 (26%) with Ph+ ALL were excluded from analysis (5-year OS probability 0.08)

1 combined OS probability for favorable/intermediate risk groups

2 patient did not enter CR

3 this group has only 12 subjects grouped as high risk despite 5-year OS probability of 0.47

**Table 3:** Identification of novel lesions by integrated molecular genetics.

	Gene/s involved	Functional consequences	Frequency		Clinical relevance
			Children	Adults	
***Genomic lesions***					
Focal deletions; rarely mutations	*IKZF1*, 7p13-p11.1	Deregulation of lymphoid differentiation	15%; >80% *BCR-ABL* pos; ~30% HR *BCR-ABL-*	7%; > 80% *BCR-ABL* +	Poor outcome
Rearrangements; interstitial Par1 deletion; mutations	*CRLF2*, Xp22.3; Yp11.3	Together with *JAK* mutations, constitutive JAK-STAT activation	5–10%;>50 DS-ALL	5–10%	Poor outcome
Mutations	*JAK1*, 1p32.3-p31.3 *JAK2*, 9p24	Constitutive JAK-STAT activation	~10% HR-*BCR- ABL*+; 18%–35% DS-ALL	-	Associated with *CRLF2*, *IKZF1,* poor outcome
Focal deletions; mutations	*CREBBP*, 16p13.3, *EP300*, 22q13.2	Impaired histone acetylation and transcriptional regulation	18% of relapsed ALL		Increased incidence at relapse; association with glucocorticoid resistance.
Focal deletions; mutations	*NT5C2,* 10q24.32	Increased dephosphorylation of nucleoside analogs	10% of relapsed ALL (also in T-ALL)		Identified only at relapse
Intrachromosomal amplification of chromosome 21	*RUNX1*, 21q22.3	Multiple copies of the *RUNX1* gene; possible secondary event	2%	-	Poor outcome
TP53 disruption	*TP53,* 17p13.1	Mutations and/or deletions	90% hypodiploid ALL 6–11% relapsed childhood ALL (also in T-ALL)	8% of ALL at onset of disease (also in T- ALL)	Poor outcome
***Novel subgroups***					
BCR/ABL-like	Causal gene not known Possible: I*GH@CRLF2, NUP214 -ABL1, EBF1-PDGFRB*, *BCR- JAK2, STRN3-JAK2, IGH@-EPOR* *Δ-CRLF2* *IKZF1 deletion*	BCR/ABL-like signature	17%	25%	Poor outcome

HR: high-risk; DS-ALL: Down syndrome ALL

**Table 4:** Summary of recurrent genetic lesions and mutations in T-ALL.

Translocations					

	Gene/s involved	Functional consequences	Frequency		Clinical relevance

			Children	Adults	

Translocation of TCR with various oncogenes	*LMO1, LMO2, TAL1, TLX1, TLX3*	Hemopoiesis deregulation, impairment of differentiation	~ 35%		No impact
t(1;14)					
t(10;14)					
t(5;14)					

t(8;14)(q24;q11)					Lymphoma-like presentation, aggressive disease/poor outcome

Del(1)(p32)	*SIL-TAL1*	Impairment of differentiation	~10%	5–10%	Not clearly established

9p deletion	*CDKN2A* and *CDKN2B*	Loss of cell proliferation control	20–30%	<1%	No impact

11q23 rearrangements	*MLL* with various partners	Disruption of HOX genes expression and of self- renewing properties of hemopoietic progenitors	~5%		Poor outcome

t(9;9)(q34;q34)	*NUP214-ABL*	*ABL* constitutive activation	6%		No impact

t(9;14)(q34;q32)	*EML1-ABL*	*ABL* constitutive activation	1%		No impact

**Mutations**					

*NOTCH1* (9q34.3)		Impairment of differentiation of and proliferation	60–70%	60–70%	Overall favorable outcome

*FBW7* (4q31.3)		Arrest of differentiation, and aberrant self renewal activity	~10%	~10–20%	Usually evaluated in combination with *NOTCH1*

*BCL11B (*14q32.2)		Loss of cell proliferation control	9%	-	Not defined

*JAK1* (1p32.3-p31.3)		Cytokine growth independence, resistance to dexamethasone-induced apoptosis, JAK signaling activation	2%	7–18%	Unfavorable outcome

*PTPN2* (18p11.3-p11.2)		Negative regulator of tyrosine kinases	6%	-	No impact

*IL7R* (5p13)		Lymphoid development	6%	-	No impact

*PHF6* (Xq26.3)		Putative tumor suppressor	5–16%	18–38%	No impact

*CNOT3* (19q13.4)		Presumed tumor suppressor	-	8%	

*RPL5* (1p22.1)		Ribosomal activity impairment	8%	-	

*RPL10* (Xq28)		Ribosomal activity impairment	8%	-	

*NT5C2* (10q24.32)		Increased dephosphorylation of nucleoside analogs	19% of relapsed ALL		Identified only at relapse

**Novel subroup**					

Early-T precursor	*Possible involved*	Specific imunophenotype and transcriptional profile miR-221, 222, 223 overexpression	~10%	~10%	Poor outcome
	*genes:*				
	*ETV6*				
	*IDH1*				
	*IDH2*				
	*DNMT3A*				
	*FLT3*				
	*NRAS*				
	*JAK3*				
	*IKZF1*				
